# Optimization of episomal reprogramming for generation of human induced pluripotent stem cells from fibroblasts

**DOI:** 10.1080/19768354.2018.1451367

**Published:** 2018-03-15

**Authors:** Jin Seok Bang, Na Young Choi, Minseong Lee, Kisung Ko, Hye Jeong Lee, Yo Seph Park, Dahee Jeong, Hyung-Min Chung, Kinarm Ko

**Affiliations:** a Department of Stem Cell Biology, School of Medicine, Konkuk University, Seoul, Korea; b Center for Stem Cell Research, Institute of Advanced Biomedical Science, Konkuk University, Seoul, Korea; c Department of Medicine, College of Medicine, Chung-Ang University, Seoul, Korea; d Research Institute of Medical Science, Konkuk University, Seoul, Korea

**Keywords:** Episomal vector, reprogramming, iPSC generation, integration-free

## Abstract

Generation of induced pluripotent stem cells (iPSCs) by defined factors (*OCT4*, *SOX2*, *C-MYC*, and *KLF4*) from various human primary cells has been reported. Human fibroblasts have been widely used as a cellular source in reprogramming studies over recent decades. The original method of iPSC generation uses retro- or lentivirus vectors that require integration of viral DNA into the target cells. The integration of exogenous genes encoding transcription factors (*OCT4*, *SOX2*, *C-MYC*, and *KLF4*) can be detected in iPSCs, raising concern about the risk of mutagenesis and tumor formation. Therefore, stem cell therapy would ideally require generation of integration-free iPSCs using non-integration gene delivery system such as Sendai virus, recombinant proteins, synthetic mRNA, and episomal vectors. Several groups have reported that episomal vectors are capable of reprogramming human fibroblasts into iPSCs. Although vector concentration and cell density are important in the episomal vector reprogramming method, optimization of this method for human fibroblasts has not been reported. In this study, we determined optimal conditions for generating integration-free iPSCs from human fibroblasts through the use of different concentrations of episomal vectors (*OCT4*/*p53*, *SOX2*/*KLF4*, *L-MYC*/*LIN28A*) and different plating cell density. We found that optimized vector concentration and cell density accelerate reprogramming and improve iPSC generation. Our study provides a detailed stepwise protocol for improved generation of integration-free iPSCs from human fibroblasts by transfection with episomal vectors.

## Introduction

Induction of pluripotency using retro- or lentiviral vectors to express reprogramming factors (*OCT4*, *SOX2*, *KLF4*, and *C-MYC*) is successful in various human cell types, including fibroblasts, urine-derived cells, and peripheral blood cells (Takahashi et al. 2007; Staerk et al. 2010; Zhou et al. 2011). Originally, viral-based methods were used, which are efficient, straightforward, reliable, and easily adoptable. However, these methods required viral vectors that integrate into the host genome, which may lead to gene mutations and tumor formation and thus limit the use of such cells in therapeutic applications. To circumvent these problems, several methods have been developed for generation of integration-free induced pluripotent stem cells (iPSCs), such as the piggyBac system, a minicircle vector, synthetic mRNA, Sendai virus, recombinant proteins, and episomal vectors (Fusaki et al. 2009; Kim et al. 2009; Woltjen et al. 2009; Yu et al. 2009; Jia et al. 2010; Okita et al. 2011).

Among integration-free methods, the episomal method is a technically simple, fast, convenient, and reproducible approach for generating iPSCs. However, episomal vectors have low reprogramming efficiency in comparison with viral vectors (Okita et al. 2010; Zhou and Zeng 2013). Furthermore, in many studies that used the episomal system, the transcription factors were delivered individually by nucleofection. However, due to differences in vector uptake by nucleofection, gene expression levels each cell are highly variable (Drozd et al. 2015).

In this study, we optimized reprogramming of human foreskin fibroblasts (BJ cells) into episomal iPSCs (Epi-iPSCs) using a stepwise protocol that uses optimized vector concentration and cell seeding density. We believe that the results described in this study can be useful in other experiments involving Epi-iPSCs generation.

## Materials and methods

### Cell culture

BJ cells were obtained from ATCC and were cultured in mouse embryonic fibroblast (MEF) medium consisting of Dulbecco's Modified Eagle's Media (DMEM; Welgene) supplemented with 10% fetal bovine serum (FBS; Corning), 0.1 mM non-essential amino acids (NEAA; Gibco), and 1% penicillin/streptomycin (P/S; Welgene). Human embryonic stem cells (hESCs) and iPSCs were plated onto Matrigel (Corning)-coated dishes and cultured in mTeSR medium (Stemcell Technology).

### Episomal vectors

Episomal vectors pCLXE-h*OCT4*/*p53*, pCLXE-h*SOX2*/*KLF4*, pCLXE-h*L-MYC*/*LIN28A* used for this study were originally described by (Okita et al. 2011) and were obtained from Addgene (Cat 27078, Cat 27080, and Cat 27077, respectively).

### Analysis of transfection rate

The optimized of Amaxa 4D-Nucleofector kit for P2 primary cell solution (Lonza) was used for transfection of BJ cells with the pmaxGFP control vector (Lonza) according to manufacturer's instructions. For each transfection, 1.5 × 10^6^ cells were pelleted by centrifugation at 13,000 rpm for 4 min at room temperature and resuspended in 82 µl of P2 primary cell solution with 18 µl of supplement 1. The pmaxGFP control vector (0–21 µg) was mixed with the BJ cells cell suspension, and transferred into a Nucleocuvette Vessel. The BJ cells were transfected using a the program setting DT-130 with 4D-Nucleofector (Lonza), plated onto Matrigel coated 6-well dishes, and cultured in MEF medium to monitor GFP expression over a period of 4 weeks.

### Flow cytometry analysis

GFP-positive cells were dissociated with 0.25% trypsin (Gibco), washed with Dulbecco's Phosphate Buffered Saline (HyClone; DPBS), and resuspended with DPBS. Fluorescence-activated cell sorting (FACS) analysis was performed using a BD Accuri C6 flow cytometer (BD Biosciences) for 4 weeks. FACS data were analyzed using the FlowJo software version 9.1 (Tree Star).

### Optimization of episomal vector concentration for generation of iPSCs

BJ cells (1.5 × 10^6^) were isolated by treatment with 0.25% trypsin and were electroporated with the pmaxGFP control vector using an Amaxa 4D-Nucleofector kit for P2 primary cell solution according to manufacturer's instructions. Each of the three episomal vectors (0–21 μg) was mixed with 82 µl of P2 primary cell solution and 18 µl of supplement 1. Transfected BJ cells were plated onto a Matrigel coated 6-well dishes in MEF medium. After 2 days post transfection MEF medium was removed and replaced with TeSR-E7 medium (Stemcell Technology). The medium was changed every day. Within 10–14 days post-transfection, Epi-iPSCs expanded to a size suitable for transfer. Before the transfer, 10 µM Y-27632 (Selleckchem) was added to the mTeSR medium. Colonies were transferred onto Matrigel coated 4-well dishes by using a 10 µl pipette tip.

### Optimization of initial plated cell number for generation of iPSCs

To generate Epi-iPSCs, 1.5 × 10^6^ BJ cells were transfected using an Amaxa 4D-Nucleofector kit for P2 primary cell solution according to manufacturer's instructions. Episomal vectors (3 μg of each: *OCT4*/*p53*, *SOX2*/*KLF4*, *L-MYC*/*LIN28A*) were mixed with 82 µl of P2 primary cell solution and 18 µl of supplement 1. A mixture of BJ cells and episomal vectors was transferred into a Nucleocuvette Vessel and electroporated with the DT-130 as the name of program. Transfected BJ cells (9 µg total for each vector) were plated onto Matrigel coated 6-well dishes (1.0 × 10^4^, 2.0 × 10^4^, 3.0 × 10^4^, 4.0 × 10^4^, 5.0 × 10^4^, 1.0 × 10^5^, 2.0 × 10^5^, and 3.0 × 10^5^ cells) in MEF medium. After 2 days post transfection MEF medium was removed and replaced with TeSR-E7 medium. The medium was changed every day. Within 10–14 days post-transfection, Epi-iPSCs expanded to a size suitable for transfer. Before the transfer, 10 µM Y-27632 was added to the mTeSR medium. Colonies were transferred onto Matrigel coated 4-well dishes by using a 10 µl pipette tip.

### RT-PCR and quantitative PCR

Total RNA was isolated using the RNeasy Kit (Qiagen) according to the manufacturer's instructions. Total RNA (1 µg) was reverse-transcribed into cDNA using the High Capacity cDNA Reverse Transcription kit (Applied Biosystems) according to the manufacturer's instructions. RT-PCR was performed using the Ex Taq Polymerase (TaKaRa) according to the manufacturer's instructions. Quantitative PCR (qPCR) was performed using the TaqMan PCR Master Mix (Thermo Scientific) and SYBR Green (Enzynomics) on a LightCycler 1536 Real-Time PCR system (Roche). All values were normalized to an endogenous *GAPDH* control.

### Alkaline phosphatase staining and immunocytochemistry

Alkaline phosphatase staining was performed using the Leukocyte Alkaline Phosphatase kit (Stemgent) according to the manufacturer's instructions. For immunocytochemistry, cells were washed with DPBS and fixed in 4% paraformaldehyde for 15 min at room temperature. The fixed cells were permeabilized with 0.5% Triton X-100 (Sigma-Aldrich) in DPBS for 10 min at room temperature and then blocked with 2% diluted bovine serum albumin (Sigma-Aldrich) in DPBS. The cells were then incubated in primary antibody solution overnight at 4°C. After washing with DPBS, the cells were incubated in secondary antibody for 1 h at room temperature.

### Genomic DNA isolation and bisulfite sequencing

To determine the DNA methylation status of Epi-iPSCs, genomic DNA was isolated using a G-spin Total DNA Extraction Kit (iNtRON). Genomic DNA (1 µg) was modified using an EpiTect Bisulfite Kit (Qiagen) according to the manufacturer's instructions. The promoter region of human *OCT4* gene was amplified by PCR. The amplified products were purified using a QIAquick Gel Extraction Kit (Qiagen) and subcloned into the TA cloning vector (Invitrogen). Individual clones were sequenced using M13 forward primers. The data were visualized and aligned using QUMA (Quantification Tool for Methylation Analysis; http://quma.cdb.riken.jp/).

### Differentiation of Epi-iPSCs

Embryoid bodies (EBs) were generated by plating Epi-iPSCs into 60 mm^2^ bacterial-grade dishes. EBs composed of approximately 2.0 × 10^6^ cells were in mTeSR medium. After 5 days, EBs were transferred to new 60 mm^2^ bacterial-grade dishes and were maintained in suspension culture in MEF medium for 14 days. For differentiation into endoderm, EBs were plated onto Matrigel coated 4-well dishes in endodermal differentiation medium consisting of RPMI 1640 (Gibco) supplemented with 2% FBS, 100 ng/µl of Activin A (PeproTech), 1% L-glutamine (Gibco), and 1% P/S for 3 weeks. For differentiation into ectoderm, EBs were plated onto Matrigel coated 4-well dishes in ectodermal differentiation medium consisting of DMEM/F12 (Corning) supplemented with 1 ml of N-2 supplements, 10 ng/ml of basic fibroblast growth factor (PeproTech), 2 µM SB431524 (Tocris), 100 ng/µl of Noggin (PeproTech), 1% L-glutamine, and 1% P/S for 3 weeks. For differentiation into mesoderm, EBs were plated onto Matrigel coated 4-well dishes in mesodermal differentiation medium consisting of KnockOut DMEM (Gibco) supplemented with 20% FBS, 100 µM ascorbic acid (Sigma-Aldrich), 1% NEAA, 1% L-glutamine, and 1% P/S for 3 weeks.

### Episomal copy number determination

After transfection, BJ cells were cultured for the indicated number of days (BJ cells-D5, Epi-iPSCs (P1), Epi-iPSCs (P15), BJ cells), and genomic DNA was extracted. To determine the number of vector copies per cell, serial dilution of the vector and genomic DNA were used to generate a standard curve. The copy number of *EBNA-1* and *FBXO15* in each Epi-iPSCs sample was determined from observed threshold cycle (Ct) values using qPCR with plasmid-specific primers.

### Whole-transcript expression arrays

Total RNA samples were prepared from hESCs, Epi-iPSCs, and negative control BJ cells using RNeasy columns (Qiagen) according to the manufacturer's instructions. The Affymetrix Whole-Transcript Expression Array processing was performed according to the manufacturer's protocol with a GeneChip Whole Transcript PLUS Reagent kit. cDNA was synthesized using the GeneChip WT (Whole Transcript) Amplification kit as described by the manufacturer. The sense cDNA was then fragmented and biotin-labeled with TdT (terminal deoxynucleotidyl transferase) using the GeneChip WT Terminal Labeling kit. Approximately 5.5 μg of labeled DNA target was hybridized to the Affymetrix GeneChip Human 2.0 ST Array at 45°C for 16 h. Hybridized arrays were washed and stained on a GeneChip Fluidics Station 450 and scanned on a GCS3000 Scanner (Affymetrix). Raw data were extracted automatically in the Affymetrix data extraction protocol using the Affymetrix GeneChip Command Console Software (AGCC).

### Teratoma formation

The Epi-iPSCs (2.0 × 10^6^) were mixed with mTeSR containing Matrigel and injected subcutaneously to dorsolateral flank of immunodeficient mice. At 8 weeks after injection, tumor were dissected and fixed in 4% paraformaldehyde. Paraffin-embedded tissue was sliced and stained with hematoxylin and eosin (Sigma-Aldrich).

## Results

### Optimization of GFP plasmid transfection in human fibroblasts

**Figure 1.**
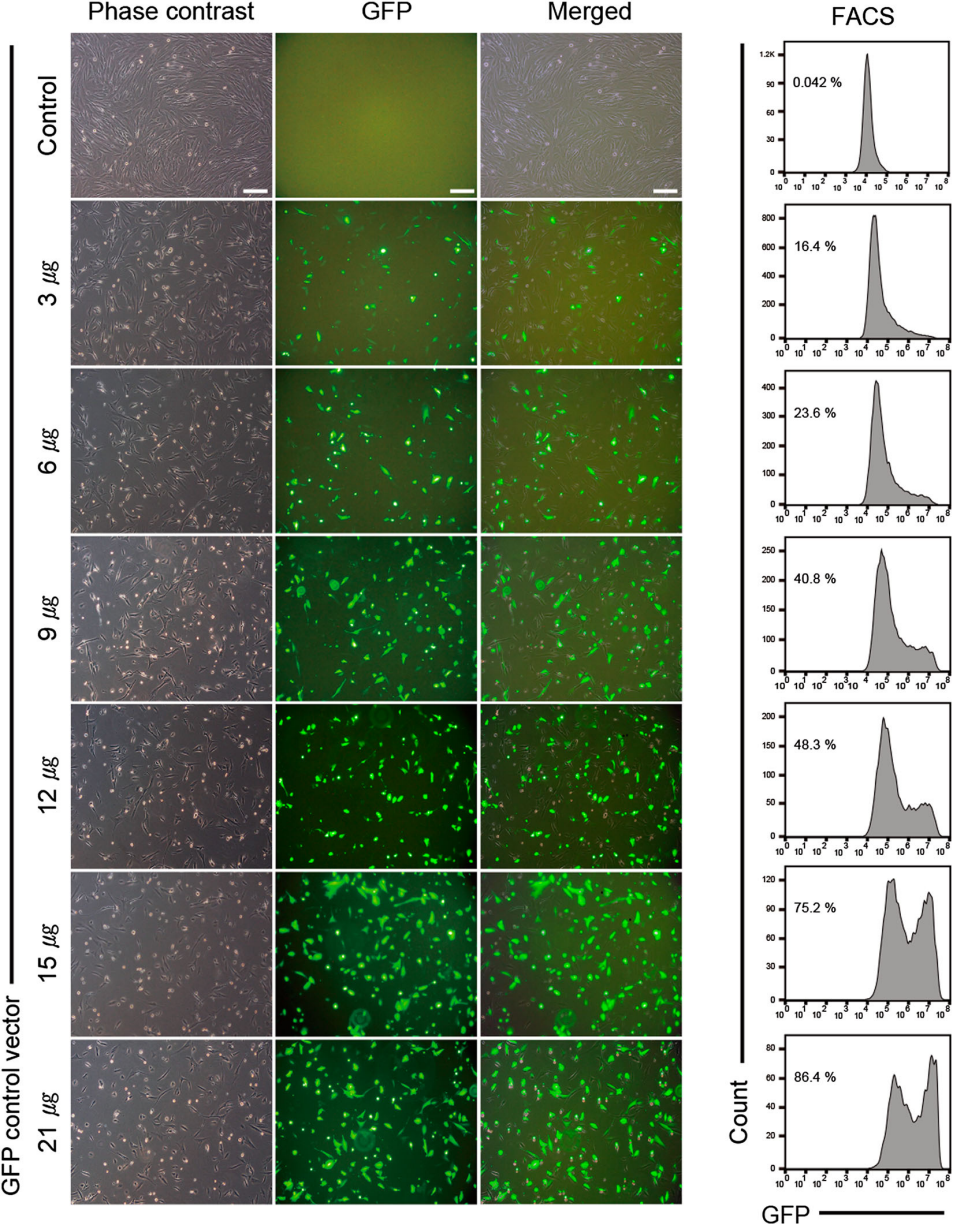
Transfection efficiency of BJ cells with an episomal vector encoding GFP. Expression of GFP after transfection was monitored by fluorescence microscopy and FACS analysis. Scale bars: 100 μm.

To optimize the amount of the vector used for transfection, we transfected 1.0 × 10^5^ BJ cells with different amounts of an episomal vector encoding GFP and monitored GFP expression over a period of 4 weeks under a fluorescence microscope (Figure 1). For quantitative analysis of transfection rate, we analyzed GFP expression using flow cytometry. We found that GFP was expressed in 16.4% of the cells at 1 week post-transfection when cells were transfected with the lowest amount of the vector (3 µg). The number of GFP-positive cells increased in a dose-dependent manner; GFP expression was detected in 86.4% of the cells transfected with the highest amount of the vector (21 µg). Our results show that increasing the amount of episomal vector improved the transfection efficiency (Figure 1). However, we found that occurrence of cell death was also increased in a dose-dependent manner (data not shown). Therefore, 21 μg was chosen as the maximum amount of three episomal vectors for optimization of iPSC generation.

### Dosage optimization of each vector for human fibroblast reprogramming

**Figure 2.**
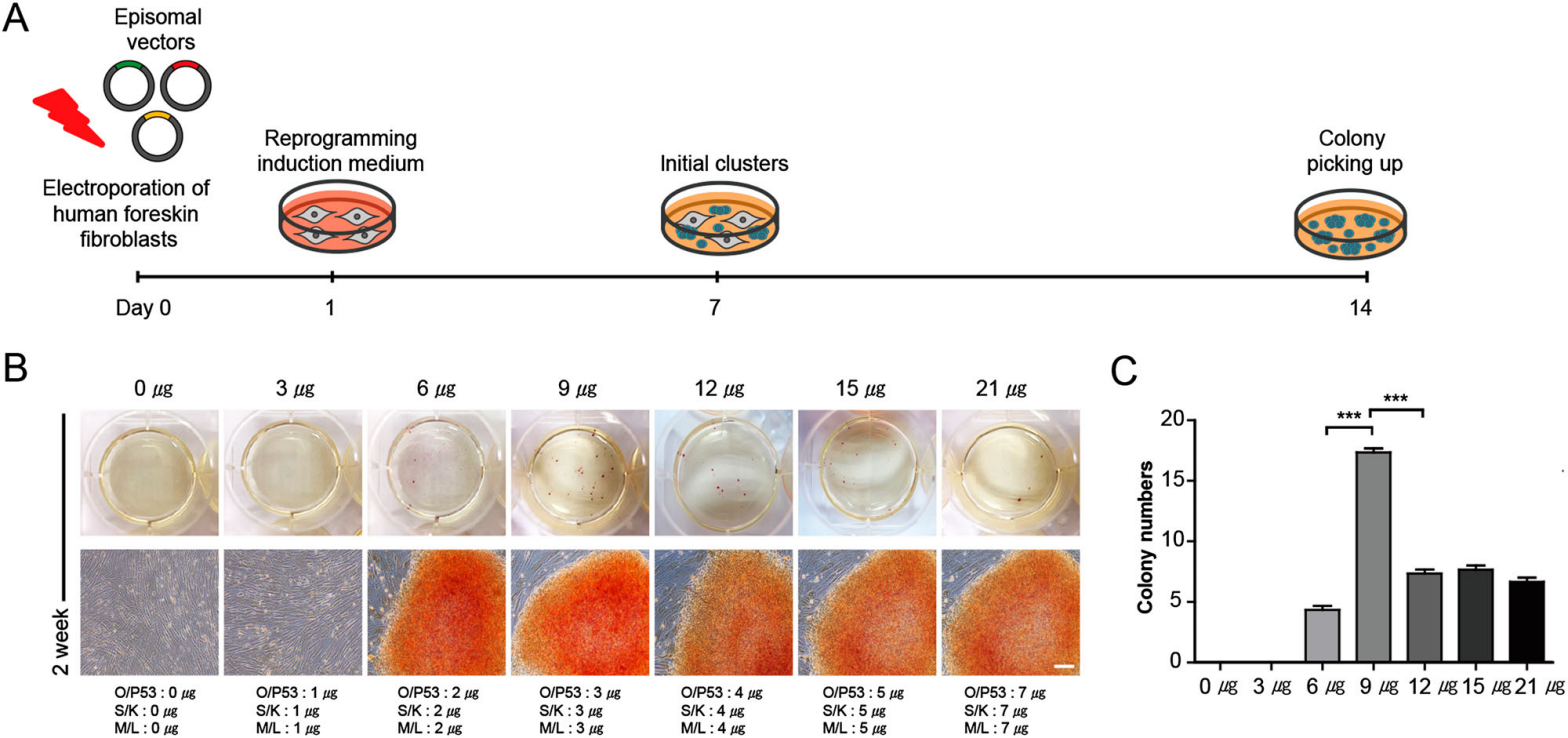
Generation of integration-free Epi-iPSCs from fibroblasts by using episomal vectors. (A) A scheme depicting the procedure. (B) The morphology of Epi-iPSCs and alkaline phosphatase staining on day 14 after transfection with 0–21 μg of each episomal vectors (*OCT4*/*p53*; O/P53, *SOX2*/*KLF4*; *S/K, L-MYC*/*LIN28A*; M/L), and established Epi-iPSCs as assessed by bright-field microscopy. Scale bar = 100 μm. (C) Number of Epi-iPSCs colonies on day 14 after transfection with different amounts of episomal vectors. The data were represented mean ± SEM and analyzed by one-way ANOVA. *** *p* < 0.001 (*n* = 3).

We optimized the method for BJ cells transfection using episomal vectors (Figure 2(A)). After 2 weeks, we observed the first Epi-iPSC clusters, which displayed typical iPSC-like morphology, and we counted the number of alkaline phosphatase–positive colonies plate formed by cells transfected with different amounts of the plasmid (0–21 µg; Figure 2(B)). We found that using a mixture of the three vectors (3 µg of each vector; total 9 µg) considerably increased reprogramming efficiency in comparison with other vector amounts (Figure 2(C)). We then picked iPSC-like colonies from each group and expanded Epi-iPSCs in mTeSR-medium for 20 passages (data not shown).

BJ cells transfected with 0 or 3 µg of episomal vectors showed no iPSC-like colonies, indicating that these two concentrations were insufficient to induce reprogramming in this setting. We further investigated the optimal concentration of episomal vector for BJ cells reprogramming. While 9 µg gave the highest transfection efficiency, an increase to 12–21 µg significantly decreased reprogramming efficiency (by ∼50%; Figure 2(C))

.

### Characterization of Epi-iPSCs

**Figure 3.**
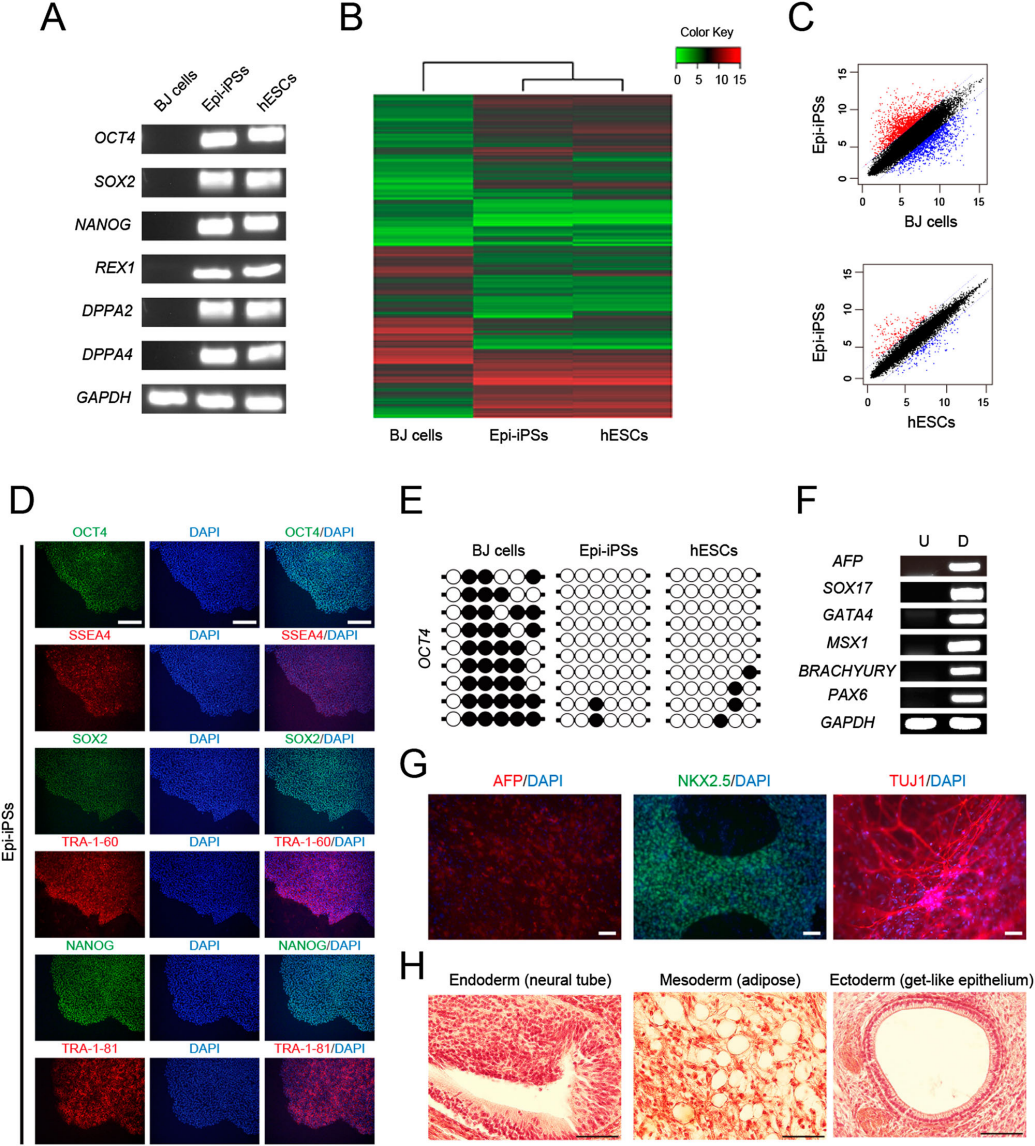
Molecular and cellular characterization of Epi-iPSCs and analysis of their *in vitro* and *in vivo* differentiation potential. (A) Expression of pluripotency markers was analyzed by RT-PCR in human fibroblasts, Epi-iPSCs, and hESCs. (B) Heat map representing the global gene expression profiles of BJ cells, Epi-iPSCs, and hESCs. Red and green colors represent high and low gene expression levels, respectively. (C) Pairwise scatter plot comparing global gene expression patterns between BJ cells, Epi-iPSCs, and hESCs. (D) Immunofluorescence microscopy images of pluripotency markers (OCT4, SOX2, NANOG, SSEA4, TRA-1-60, and TRA-1-81) in Epi-iPSCs. Scale bars = 20 μm. (E) DNA methylation analysis of the *OCT4* promoter by bisulfite sequencing. Each line represents a separate clone. Open and filled circles represent methylated and unmethylated CpGs, respectively. (F) RT-PCR analyses of *in vitro* differentiation markers for the three germ layers in Epi-iPSCs (undifferentiated, U) and differentiated cells (D). (G) *In vitro* differentiation of Epi-iPSCs into endoderm-like cells (AFP), mesoderm-like cells (NKX2.5), and ectoderm-like cells (MAP2). Scale bars = 100 μm. (H) Teratoma tissue sections were stained with hematoxylin and eosin. Shown is a teratoma containing a gut-like epithelial (endoderm), adipose (mesoderm), neural tube (ectoderm). Scale bars = 100 μm.

We examined whether the iPSC-like colonies possessed morphological, molecular, and functional properties typical of hESCs. RT-PCR analysis showed the activation typical pluripotency genes (*OCT4*, *SOX2*, *NANOG*, *REX1*, *DPPA2*, and *DPPA4*) in iPSC-like cells (Figure 3(A)). To evaluate the reprogramming status of Epi-iPSCs at high resolution, we analyzed their global gene expression profile using microarray. Heat map analysis indicated that Epi-iPSCs closely clustered together with hESCs, but were clearly separated from BJ cells. Specifically, genes known to be involved in pluripotency such as *OCT4*, *SOX2*, *NANOG*, and *LIN28A*, were up-regulated in both Epi-iPSCs and hESCs compared to BJ cells. Conversely, genes typically expressed in fibroblasts, such as *COL3A1*, *DKK1*, and *IL1R1*, were down-regulated in both Epi-iPSCs and hESCs compared to BJ cells (Figure 3(B)). Flow cytometry analysis revealed that Epi-iPSCs were clustered together with hESCs, but were clearly distinct from BJ cells (Figure 3(C)). We also examined the induction of pluripotency at the protein level. The pluripotency markers OCT4, SOX2, NANOG, SSEA4, TRA-1-60, and TRA-1-81 were expressed in both Epi-iPSCs and hESCs as determined by immunostaining (Figure 3(D)). We checked the DNA methylation status of the pluripotency marker *OCT4*. Notably, the promoter region of *OCT4* was unmethylated in Epi-iPSCs, to a level similar to that in control hESCs (Figure 3(E)). Finally, we assessed the differentiation ability of Epi-iPSCs by inducing their differentiation *in vitro* into endoderm, ectoderm, and mesoderm. RT-PCR confirmed the expression of three germ layer marker genes in Epi-iPSCs and differentiated cells. *GAPDH* was used as a positive control (Figure 3(F)). Immunocytochemistry analysis confirmed that Epi-iPSCs expressed the endoderm marker *AFP*, mesoderm *NKX2.5*, and ectoderm *MAP2* (Figure 3(G)). To investigate *in vivo* differentiation ability of Epi-iPSCs, we transplanted them subcutaneously into NOD/SCID mice. After 8 weeks, we observed teratoma formation containing three germ layers, endoderm, mesoderm, and ectoderm tissues (Figure 3(H)).

### Cell density–dependent reprogramming for Epi-iPSC generation

**Figure 4.**
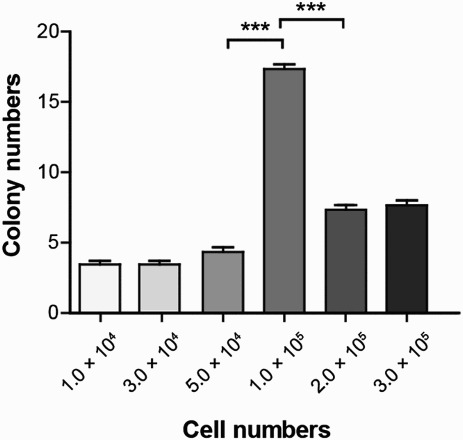
Number of Epi-iPSC colonies as a function of BJ cell density. Representative numbers of colonies at day 14 post-transfection are shown. 1.0 × 10^4^ to 3.0 × 10^5^ BJ cells were seeded at the indicated cell density per well after transfection with 9 μg of the same episomal vectors. The data were represented mean ± SEM and analyzed by one-way ANOVA. *** *p* < 0.001 (*n* = 3).

Another technical obstacle that generally arises during iPSC generation is cell death caused by electroporation used for transfection. To overcome the reprogramming failure caused by cell death, we generated Epi-iPSCs from BJ cells plated at different cell density and transfected with 3 μg of each vector. The initial Epi-iPSCs clusters were observed around 2 weeks after transfection (data not shown). A density of 1.0 × 10^5^ cells per well in a 6-well dish was most efficient for generating Epi-iPSCs (Figure 4). Taken together, our data indicate that, to achieve high-efficiency BJ cells reprogramming, the optimal concentration is 3 µg of each of the three episomal vectors encoding *OCT4*/*P53*, *SOX2*/*KLF4*, and *L-MYC*/*LIN28A* and a cell density of 1.0 × 10^5^.

## Discussion

Generation of iPSCs through an integration-free approach is still less efficient than another viral-based reprogramming system (Drozd et al. 2015). However, integration-free iPSCs hold great potential for cell therapy and regenerative medicine (Wen et al. 2016). In this study, we generated functional and expandable Epi-iPSCs using a non-viral integration-free system. As efficient pluripotency gene transfer is a condition for the successful generation of Epi-iPSCs, we determined the optimal conditions for delivery of episomal vectors. First, we carried out pmaxGFP control vector expression experiments to find out which transfection conditions lead to highest expression. It has been reported that increasing plasmid concentration increased transfection efficiency, as evidenced by an increase in the proportion of GFP positive cells (Carlotti et al. 2004; Palchetti et al. 2017). However, in our experience, the survival rate of BJ cells after electroporation was less than 50% (data not shown); increasing the amount of plasmid DNA during transfection likely increases cell death.

We attempted to develop a novel reprogramming system by using episomal vectors. To further examine the effects of different vector concentrations, we checked the concentrations of vectors ranging from 0 to 21 µg, and found that 9 µg resulted in the highest transfection efficiency. Similarly, 9 µg of episomal vectors in iPSC generation has been shown to be the optimal amount in urine-derived cells (Li et al. 2016). However, we found that reprogramming efficiency decreased when the total vector concentration was too high. Previous studies have shown that cell density is critical for reprogramming (Zhao et al. 2015; Sia et al. 2016). Thus, to optimize cell density, we plated BJ cells transfected with 9 µg episomal vectors onto Matrigel coated 6-well dishes at a density ranging from 1.0 × 10^4^ to 3.0 × 10^5^ cells per well. We found that a cell density of 1.0 × 10^5^ cells per well of 6-well dish allows achieving the highest efficiency of BJ cells reprogramming.

Finally, we found that transfection with 9 µg of episomal vectors was sufficient to generate iPSCs, and a cell density of 1.0 × 10^5^ cells per well of 6-well dish was adequate to generate them within 2 weeks. We described here an optimized approach for the generation of Epi-iPSCs from BJ cells. To the best of our experience, this is the easiest and fastest method to generate Epi-iPSCs. Therefore, our optimization of the transfection conditions would be helpful to the generation of integration-free human iPSCs.
